# A *Dictyostelium *chalone uses G proteins to regulate proliferation

**DOI:** 10.1186/1741-7007-7-44

**Published:** 2009-07-27

**Authors:** Deenadayalan Bakthavatsalam, Jonathan M Choe, Nana E Hanson, Richard H Gomer

**Affiliations:** 1 Department of Biochemistry and Cell Biology, MS-140, Rice University, Houston, TX, USA

## Abstract

**Background:**

Several studies have shown that organ size, and the proliferation of tumor metastases, may be regulated by negative feedback loops in which autocrine secreted factors called chalones inhibit proliferation. However, very little is known about chalones, and how cells sense them. We previously identified two secreted proteins, AprA and CfaD, which act as chalones in *Dictyostelium*. Cells lacking AprA or CfaD proliferate faster than wild-type cells, and adding recombinant AprA or CfaD to cells slows their proliferation.

**Results:**

We show here that cells lacking the G protein components Galpha8, Galpha9, and Gbeta proliferate faster than wild-type cells despite secreting normal or high levels of AprA and CfaD. Compared with wild-type cells, the proliferation of *galpha8^-^*, *galpha9^- ^*and *gbeta^- ^*cells are only weakly inhibited by recombinant AprA (rAprA). Like AprA and CfaD, Galpha8 and Gbeta inhibit cell proliferation but not cell growth (the rate of increase in mass and protein per nucleus), whereas Galpha9 inhibits both proliferation and growth. *galpha8^- ^*cells show normal cell-surface binding of rAprA, whereas *galpha9^- ^*and *gbeta^- ^*cells have fewer cell-surface rAprA binding sites, suggesting that Galpha9 and Gbeta regulate the synthesis or processing of the AprA receptor. Like other ligands that activate G proteins, rAprA induces the binding of [^3^H]GTP to membranes, and GTPgammaS inhibits the binding of rAprA to membranes. Both AprA-induced [^3^H]GTP binding and the GTPgammaS inhibition of rAprA binding require Galpha8 and Gbeta but not Galpha9. Like *aprA^- ^*cells, *galpha8^- ^*cells have reduced spore viability.

**Conclusion:**

This study shows that Galpha8 and Gbeta are part of the signal transduction pathway used by AprA to inhibit proliferation but not growth in *Dictyostelium*, whereas Galpha9 is part of a differealnt pathway that regulates both proliferation and growth, and that a chalone signal transduction pathway uses G proteins.

## Background

Many tissues or organs have an inherent property of growing to a particular size [1]. In some cases, it appears that size regulation is mediated by secreted factors called chalones, which, as part of a negative feedback loop, repress the proliferation of the cells that secrete the chalones, so that when there is a high number or density of the cells, the corresponding high concentration of the chalone slows proliferation [2-4]. For instance, myostatin, a member of the TGFβ superfamily, is secreted by muscle cells and negatively regulates myoblast proliferation, and thus controls muscle size in a body [5]. Interestingly, many primary tumors appear to secrete factors that repress the proliferation of the metastatic cells, but the factors are unknown [6,7]. Although some chalones have been identified, much remains to be understood about their signal transduction pathways.

*Dictyostelium *is a unicellular eukaryote and an excellent model system to study the regulation of proliferation and growth. We previously identified two proteins secreted by *Dictyostelium*, AprA and CfaD, which appear to act like chalones. Cells lacking AprA or CfaD have an abnormally high proliferation rate, and as a result when cells reach a high cell density they have less mass and protein per nucleus [3,4]. When starved, *aprA^- ^*and *cfaD^- ^*cells form spores that have poor viability compared with wild-type spores, or if cultures are maintained after cells reach saturation, the cells then die relatively quickly [3,4]. This suggests that *Dictyostelium *cells use chalones to slow proliferation at high cell density (when they are probably about to overgrow their food supply and starve) so that the cells will have more nutrient reserves. Overexpression of either AprA or CfaD, or adding either recombinant AprA (rAprA) or recombinant CfaD (rCfaD) to cultures, slows cell proliferation [3,8]. *Dictyostelium *cells have saturable cell-surface high-affinity binding sites for AprA, suggesting the presence of an AprA signal transduction pathway [8].

A common type of signal transduction pathway in eukaryotes involves G proteins [9,10]. Upon activation, G protein-coupled receptors induce the Gα subunit of the heterotrimeric G protein complex to release GDP and bind GTP, and induce dissociation of the G protein complex to generate active α and βγ subunits. These further activate other downstream effectors to trigger intracellular processes [11]. Binding of GTP to the plasma membrane is thus increased in the presence of ligand, and this has been observed for the G protein coupled cAMP receptors in *Dictyostelium *[12]. Conversely, treating membranes with GTPγS, a non-hydrolyzable GTP analog generally reduces affinity of the receptors for the ligand, and this has also been observed for the cAMP receptor in *Dictyostelium *[13].

The *Dictyostelium *genome appears to encode 12 Gα, 2 Gβ, and 1 Gγ subunits [14], and 55 G-protein-coupled receptors [15]. Of the 12 Gα subunits, 8 have been characterized, and all characterized Gα subunits and the single characterized Gβ are expressed in vegetative cells, with the possible exception of Gα5 [16].

In this study we investigated whether AprA uses a G protein mediated signaling to repress proliferation. We found that the proliferation of *gα8*^-^, *gα9*^-^, and *gβ*^- ^cells was faster than wild-type cells, and compared with wild-type their proliferation was only weakly inhibited by rAprA. Cells lacking Gα9 and Gβ have a reduced number of cell-surface rAprA binding sites, suggesting that Gα9 and Gβ regulate the availability or processing of the AprA receptor. rAprA induces GTP binding to membranes, and this process requires Gα8 and Gβ. In addition, GTPγS inhibits the binding of AprA to membranes, and this also requires Gα8 and Gβ. These data suggest a key role of Gα8 and Gβ in the AprA signaling pathway that regulates proliferation in *Dictyostelium*.

## Methods

### Cell culture

Wild-type Ax2, *aprA*^- ^strain DB60T3–8 [4], *cfaD*^- ^strain DB27C-1 [3], *crlA^- ^*strain JH557 [17], *gα1*^- ^strain DBS02306088 (the DBS numbers are the Dictybase strain identifiers) [18], *gα2*^- ^strain DBS0236094 [19], *gα3*^- ^strain DBS0235986 [20], *gα4*^- ^strain DBS0235984 [21], *gα5*^- ^strain DBS0236451 [22], *gα7*^- ^strain DBS0236106 [23], *gα8*^- ^strain DBS0236107 [23], *gα9*^- ^strain DBS0236109 [24], and *gβ*^- ^strain DBS0236531 [25] cells were grown in shaking culture as previously described [3]. The different transformant strains were produced in different labs at different times, and have different wild-type backgrounds. However, we have examined the proliferation rate of a large variety of different 'wild type' strains, including different sources of Ax2, Ax3, and Ax4, and we found that all of them have similar proliferation rates and sensitivities to rAprA (data not shown). Proliferation assays were done following [4,26]. Measurements of cell mass and protein, and counts of nuclei were done following [4] using cells harvested at 5 × 10^6^/ml with the exception that for nuclei counts, cells were fixed with 4% paraformaldehyde (Sigma Aldrich, St Louis, MO, USA) in PBS for 45 min and were then washed with PBS/0.1% Tween-20 for 15 min before staining with DAPI. Spore viability assays were done following [4].

### Purification and quantification of rAprA, and proliferation inhibition assay

His/Myc tagged rAprA was expressed and purified as described in [3]. Quantification of rAprA and the effect of exogenous rAprA on proliferation were done following [8]. To measure the amount of extracellular AprA and CfaD, conditioned growth medium was collected from cells at a density of 5 × 10^6 ^cells/ml following [8]. SDS-polyacrylamide gels of aliquots of the conditioned growth medium were silver stained to check for the amount of proteins in the samples. For the proliferation inhibition assays, cells were grown to 2 × 10^6^cells/ml, and resuspended in HL5 media to 5 × 10^5 ^cells/ml. We then added rAprA to 300 ng/ml, or an equal volume of buffer, and after 12 h cells were counted. Proliferation inhibition was defined as the percent decrease in cell density caused by rAprA compared with buffer control.

### Recombinant AprA binding assay

Binding of rAprA to cells was carried out following [8], where cells were incubated with different concentrations of rAprA at 4°C on an end-to-end tumbler for 10 min.

### Effect of GTPγS on rAprA binding to membranes

To determine the effect of GTPγS on the binding of rAprA to membranes, cells were washed twice with ice-cold HL5 and resuspended to 5.0 × 10^6 ^cells in 485 μl of ice-cold HL5. Either 5 μl of PBM (20 mM KH_2_PO_4_, 0.01 mM CaCl_2_, 1 mM MgCl_2_, pH 6.1 with KOH) or 5 μl of 10 mM GTPγS (Sigma) in PBM was added to cells. The cells were then lysed by passing the cell suspension through a Cameo 17N 5 μm pore size syringe filter (GE Osmonics, Minnetonka, MN, USA) into ice-chilled Eppendorf tubes containing 150 ng of rAprA in 10 μl of ice-cold HL5. The tubes were mixed on an end-to-end rotor at 4°C. After 10 min, the crude membranes were collected by centrifugation at 17,000 × g for 10 min at 4°C. The pellet of membranes was gently washed (without resuspending the pellet) with 500 μl of ice-cold HL5 and the pellet was collected by centrifugation at 17,000 × g for 1 min at 4°C. The supernatant was removed and the membranes were resuspended in 50 μl of SDS sample buffer and heated at 95°C for 5 min. The amount of rAprA bound to the membranes was analyzed following [8], loading 5 μl of sample per lane.

### Effect of recombinant AprA on GTP binding to membranes

Binding of 5.1 Ci/mmol [^3^H]GTP (GE Healthcare, UK) to membranes was measured following [27] with the following modification. Eppendorf tubes containing 100 μl of binding reaction (500 nM [^3^H]GTP, 5 mM ATP, 25 mM MgCl_2 _in ice-cold PBM) with or without 60 ng of rAprA were chilled on ice. To exclude non-specific binding, duplicate tubes additionally contained 0.1 mM GTP. Cells were collected and washed as described above and were resuspended to 5 × 10^7 ^cells in 1 ml of ice-cold PBM. The resuspended cells were then lysed by passing through Cameo 17N 5 μm pore size syringe filters (GE Osmonics). To initiate binding, 100 μl of lysed cells was added to the binding reaction and incubated on ice for 10 min. Membranes were collected by centrifugation at 17,000 × g for 5 min at 4°C. The pellet of membranes was gently washed without resuspending the pellet with 1 ml of ice-cold PBM, and the pellet was collected by centrifugation at 17,000 × g for 1 min at 4°C. The pellet was then dissolved in 100 μl of 1% SDS and mixed with 10 ml of Bio safe II counting cocktail (Research Products International, Mount Prospect, IL, USA). The amount of [^3^H]GTP was determined using a scintillation counter (Beckman Coulter, Fullerton, CA, USA). The counts from the tubes with 0.1 mM unlabeled GTP were subtracted from the experimental tube counts to obtain the counts for bound [^3^H]GTP.

### Statistics

All statistics were done with Prism (GraphPad software, San Diego, CA, USA). One-way ANOVA using Dunnett's or Tukey's tests were done to compare multiple values, and paired t tests were done to compare paired values. Significance was defined as *P *< 0.05.

## Results

### Gα8, Gα9, and Gβ potentiate AprA signal transduction

**Figure 1:**
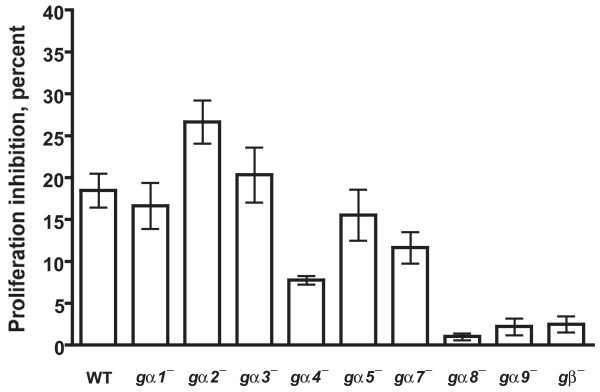
**The effect of recombinant AprA (rAprA) on cell proliferation**. Cells were grown in the presence or absence of rAprA for 12 h, and the percent decrease in cell density caused by rAprA was calculated. When compared with the inhibition of wild-type (WT) cell proliferation, the inhibition of *gα1*^- ^, *gα2*^-^, *gα3*^-^, *gα5*^-^, and *gα7*^- ^cells is not significant with *P *> 0.05 (one-way ANOVA, Dunnett's test), whereas the inhibition of *gα4*^- ^is significant with *P *< 0.01, and the inhibition of *gα8*^-^, *gα9*^-^, and *gβ*^- ^cells is significant with *P *< 0.001. The inhibition values for *gα8*^-^, *gα9*^-^, and *gβ*^- ^cells are not significantly different between each other with *P *> 0.05 (one-way ANOVA, Tukey's test), but the inhibition of *Gα4*^- ^cells is significantly different from that of *gα8*^-^, *gα9*^-^, and *gβ*^- ^cells with *P *< 0.01 (one-way ANOVA, Dunnett's test). Values are the mean ± s.e.m. from at least four separate experiments.

We previously found that *Dictyostelium *cells are able to bind AprA, suggesting that AprA is sensed by a cell surface receptor [8]. Since the *Dictyostelium *genome encodes many G protein coupled receptors, it is possible that G proteins could be involved in AprA signal transduction. To examine this possibility, we analyzed the effect of rAprA on the proliferation of *gβ*^- ^cells. When treated with rAprA, the proliferation of wild-type cells was inhibited by ~17% (Figure 1), which is similar to what we have previously observed [8], whereas the proliferation of *gβ*^- ^cells was only minimally inhibited. This suggests that AprA signal transduction may be defective in *gβ*^- ^cells. As with wild-type cells, rAprA inhibited the proliferation of *gα1*^- ^, *gα2*^-^, *gα3*^-^, *gα5*^-^, and *gα7*^- ^cells but had little effect on the proliferation of *gα8*^- ^and *gα9*^- ^cells (Figure 1). Although the proliferation of *gα4*^- ^cells was partially inhibited by rAprA, the inhibition was significantly higher than that for *gα8*^-^, *gα9*^-^, and *gβ*^- ^cells (Figure 1). Together, the data suggest that gα8, gα9 and Gβ strongly potentiate AprA signaling.

### Gα8, Gα9, and Gβ slow cell proliferation

**Figure 2:**
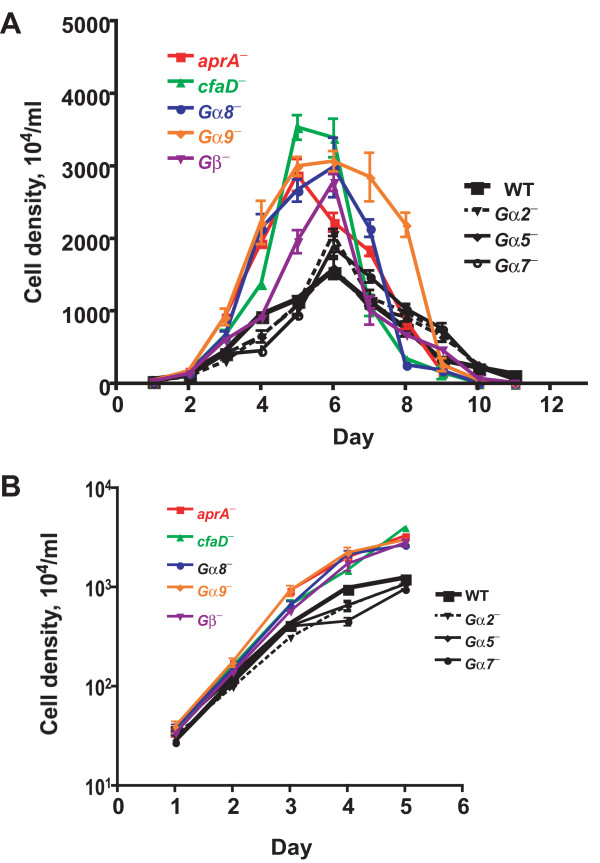
**Cells lacking Gα8, Gα9 or Gβ proliferate faster than wild-type cells**. **(A)** Cells were diluted to 2 × 10^5^cells/ml in HL5 and the cell density was measured daily. The graph shows means ± s.e.m. from three independent experiments. The differences between the maximum cell density attained by wild-type cells and *aprA*^-^, *cfaD*^-^, *gα8*^-^, *gα9*^-^, and *gβ*^- ^mutants are significant with *P *< 0.05, whereas the difference between the wild-type maximum density and the *gα2*^-^, *gα5*^-^, and *gα7*^- ^maximum densities are not significant (one-way ANOVA, Dunnett's test). **(B)** The data from the first five days was plotted using a log scale for the density. The absence of error bars indicates that the error was smaller than the plot symbol.

Cells lacking AprA proliferate faster than wild-type cells, and exogenous AprA slows proliferation [3,4]. Cells deficient in AprA signal transduction thus may also have altered proliferation. Proliferation curves were done to determine whether cells lacking G proteins had altered proliferation. We found that wild-type cells proliferated similarly to what we have previously seen [3] (Figure 2A). The proliferation of *gα2*^- ^, *gα5*^-^, and *gα7*^- ^cells was similar to the wild-type cells, while *gα8*^-^, *gα9*^-^, and *gβ*^- ^cells proliferated faster and reached higher cell densities, similar to *aprA^- ^*and *cfaD^- ^*cells (Figure 2A). At low cell densities, where the levels of secreted factors such as AprA or CfaD are low [3,4], all the cell lines have doubling times between 10 and 14 h, with the proliferation of *aprA*^-^, *cfaD*^-^, *gα8*^-^, *gα9*^-^, and *Gβ*^- ^cells significantly faster than wild-type (Figure 2A and 2B, and Table 1). At high cell densities, where the levels of AprA and CfaD are high, the doubling time of wild-type cells slowed to ~35 h (Table 1). The doubling times of *aprA*^-^, *cfaD*^-^, *gα8*^-^, and *gα9*^- ^were significantly shorter, with the doubling time of *gα8*^- ^cells at high cell density similar to the doubling time of the wild-type cells at low cell density (Figure 2B and Table 1). We previously observed that after cells reached stationary phase and stopped proliferating, *aprA^- ^*and *cfaD^- ^*cells died faster than wild-type cells [3,4]. In the growth curves presented here, we invariably saw that at days 10 and 11 there were still viable wild-type, *gα2^-^, gα5^-^*, and *gα7^- ^*cells, but the *aprA^-^*, *cfaD^-^*, and *gα8^- ^*cells appeared to be all dead at day 10, and there were few if any live *gα9^- ^*and *Gβ^- ^*cells at day 11. Together, the data suggest that, like AprA and CfaD, Gα8, Gα9, and Gβ slow cell proliferation, and cells lacking these proteins die off faster than wild-type after cells reach stationary phase.

**Table 1:** The effect of G proteins on the doubling time of cells

	**Doubling time, hours**	
**Cell type**	**Low density**	**High density**
Wild-type	13.0 ± 0.6	35.4 ± 4.3
*aprA^-^*	9.7 ± 0.1**	20.7 ± 0.4*
*cfaD^-^*	10.9 ± 0.4*	17.7 ± 0.1**
*gα2^-^*	13.9 ± 0.1	34.9 ± 3.3
*gα5^-^*	12.2 ± 0.5	34.6 ± 1.3
*gα7^-^*	12.2 ± 0.9	28.6 ± 5.4
*gα8^-^*	10.9 ± 0.3*	13.4 ± 0.2**
*gα9^-^*	10.1 ± 0.3**	18.3 ± 1.5**
*gβ^-^*	11.1 ± 0.4*	24.1 ± 3.0

Doubling times were calculated using the data from Figure 2. Low density doubling times were calculated for cells in the range of 2 × 10^5 ^to 5 × 10^6 ^cells/ml. High density doubling times were calculated for cells in the range of 5 × 10^6 ^to 2 × 10^7 ^cells/ml. All values are means ± SEM's from three or more independent assays. A * indicates a statistically significant difference with p < 0.05; ** indicates p < 0.01, comparing the value to the wild type value (1-way ANOVA, Dunnett's test).

### Gα8 and Gβ do not affect growth, while Gα9 slows growth

Cell growth, defined as the rate of increase in mass or protein content per cell (or per nucleus if cells are multinucleate), can be regulated separately from proliferation [28-32]. We previously observed that AprA and CfaD do not affect the mass or protein content of cells [3,4]. In contrast, compared with wild-type, *gα8^-^*, *gα9^-^*, and *gβ^- ^*cells had more mass per cell, and *gα9^- ^*and *gβ^- ^*cells had more protein per cell (Table 2). Compared with wild type, *aprA^- ^*and *cfaD^- ^*cells tend to be multinucleate [3,4]. Similarly, *gα8^- ^*and *gβ^- ^*cells also tended to be multinucleate (Table 2). Both *aprA^- ^*and *cfaD^- ^*cells have less mass and protein per nucleus [3,4]. In contrast, we observed that compared with wild-type, *gα9^- ^*cells have more mass and protein per nucleus, whereas *gα5^- ^*cells have less protein per nucleus (Table 2). Compared with wild-type cells, *gα8^-^*, *gα9^-^*, and *gβ^- ^*cells had a greater increase in mass and protein per cell per hour, and *gα8^- ^*and *gβ^- ^*cells had a greater increase in nuclei per cell per hour (Table 3). However, when normalized to nuclei, only *gα9^- ^*cells had a greater increase in mass or protein per hour. Together, the data indicate that although *gα8^- ^*and *gβ^- ^*cells proliferate faster than wild-type cells, their growth per nucleus is not statistically different from wild-type. Conversely, *gα9^- ^*cells have a greater growth per nucleus than wild-type cells.

**Table 2:** The effect of G proteins on the mass and protein content of cells

	**Per 10^7 ^cells**		**Percent of cells with n nuclei**			**Per 10^7 ^nuclei**		
**Cell type**	**Mass, mg**	**Protein, mg**	**1**	**2**	**3 +**	**Nuclei/100 cells**	**Mass, mg**	**Protein, mg**
Wild-type	10.8 ± 0.6	0.32 ± 0.01	76 ± 3	21 ± 2	3 ± 1	129 ± 5	8.4 ± 0.6	0.25 ± 0.01
*gα2^-^*	10.7 ± 0.3	0.33 ± 0.01	72 ± 3	22 ± 2	6 ± 2	134 ± 6	8.0 ± 0.5	0.25 ± 0.02
*gα5^-^*	12.1 ± 0.5	0.25 ± 0.01	59 ± 6	31 ± 4	11 ± 3	154 ± 8	7.9 ± 0.6	0.16 ± 0.01*
*gα7^-^*	8.8 ± 0.3	0.28 ± 0.01	68 ± 6	25 ± 4	6 ± 2	140 ± 9	6.3 ± 0.5	0.20 ± 0.01
*gα8^-^*	15.5 ± 0.1**	0.40 ± 0.02	56 ± 5*	30 ± 4	14 ± 2**	166 ± 8*	9.3 ± 0.5	0.24 ± 0.02
*gα9^-^*	16.3 ± 1.5**	0.42 ± 0.01*	78 ± 4	20 ± 3	2 ± 1	124 ± 4	13.2 ± 1.3**	0.34 ± 0.02*
*gβ^-^*	15.8 ± 1.3**	0.47 ± 0.05**	52 ± 5*	32 ± 2	17 ± 2**	180 ± 9**	8.8 ± 0.9	0.26 ± 0.03

The mass and protein content of cells was measured as described in the Materials and Methods, and the percent of cells with 1, 2, 3, or more nuclei was measured by counts of DAPI-stained cells. After calculating the average number of nuclei per 10^7 ^cells, the mass and protein per 10^7 ^nuclei was calculated. All values are means ± SEM's from three or more independent assays. A * after a value indicates that the value was significantly different from the wild-type value with p < 0.05; ** indicates p < 0.01(1-way ANOVA, Dunnett's test).

**Table 3:** The effect of G proteins on the mass and protein increase of cells

	**Per 10^7 ^cells per hour**			**Per 10^7 ^nuclei per hour**	
**Cell type**	**Mass, mg**	**Protein, μg**	**Nuclei, × 10^-5^**	**Mass, mg**	**Protein, μg**

Wild-type	0.83 ± 0.06	24 ± 1	9.9 ± 0.6	0.64 ± 0.05	19 ± 1
*gα2^-^*	0.77 ± 0.02	24 ± 1	9.6 ± 0.4	0.57 ± 0.03	18 ± 1
*gα5^-^*	0.99 ± 0.06	21 ± 1	12.6 ± 0.8	0.64 ± 0.05	13 ± 1
*gα7^-^*	0.72 ± 0.06	23 ± 2	11.5 ± 1.1	0.51 ± 0.05	16 ± 2
*gα8^-^*	1.43 ± 0.04**	37 ± 2**	15.3 ± 0.8**	0.86 ± 0.05	22 ± 2
*gα9^-^*	1.61 ± 0.15**	42 ± 2**	12.2 ± 0.5	1.30 ± 0.13**	33 ± 2**
*gβ^-^*	1.42 ± 0.13**	42 ± 4**	16.2 ± 1.0**	0.79 ± 0.08	23 ± 3

The mass, protein, and nuclei number values shown in Table 2 were divided by the observed high density doubling times from Table 1 (the mass, protein, and nuclei numbers were measured in this density range) to obtain the approximate increases in mass, protein, and nuclei per hour. All values are means ± SEM's from three or more independent assays. A ** indicates that the value was significantly different from the wild-type value with p < 0.01 (1-way ANOVA, Dunnett's test).

### gα8^-^, gα9^- ^and gβ^- ^cells proliferate rapidly despite normal levels of AprA and CfaD

**Figure 3:**
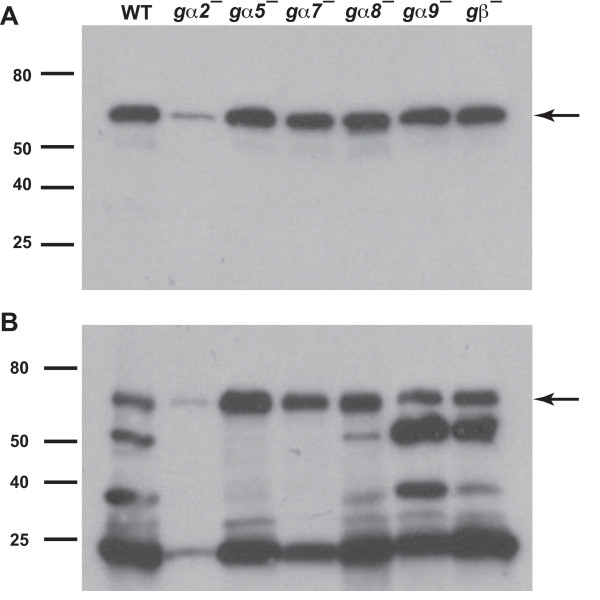
**The accumulation of extracellular AprA and CfaD**. The indicated cell types were grown in HL5 and conditioned growth media were collected. Western blots of the conditioned growth media were stained with anti-AprA antibodies **(A) **or anti-CfaD antibodies **(B)**. The arrow in A indicates the 60 kDa AprA band, and the arrow in B indicates the 62 kDa CfaD band. The lower molecular mass bands stained in B are breakdown products of CfaD [3]. Data are representative of three independent experiments.

The rapid proliferation of *gα8*^-^, *gα9*^-^, and *gβ*^- ^cells could be due to low levels of extracellular AprA or CfaD. To check this, we stained Western blots of conditioned growth media for AprA and for CfaD. Silver-stained SDS polyacrylamide gels showed that the protein profile of the conditioned growth media, and approximate amounts of the protein in the different bands, was consistently the same for all strains except for *gα2^-^*, where there appeared to be less protein in all the bands despite the fact that the starting and final density of the cells in the *gα2^- ^*cultures was very similar to wild-type (data not shown). Wild-type cells accumulated extracellular AprA to levels (~165 ng/ml) similar to what we previously observed [8] (Figure 3A and data not shown). The amount of AprA accumulated by *gα5*^-^, *gα7*^-^, *gα8*^-^, *gα9*^-^, and *gβ*^- ^cells was similar to that of wild-type cells, whereas *gα2*^- ^cells had lower (33 ng/ml) levels of AprA (Figure 3A). Wild-type cells accumulated 15 ng/ml of CfaD (Figure 3B and data not shown), again similar to what we previously observed [3]. *gα5*^-^, *gα7*^-^, *gα8*^-^, *gα9*^-^, and *gβ*^- ^cells accumulated similar or higher levels of CfaD, while *gα2*^- ^cells accumulated less (2 ng/ml) CfaD. Together, the data indicate that *gα2*^- ^cells accumulate less extracellular protein, including AprA and CfaD, than wild-type cells, and that the rapid proliferation of *gα8*^-^, *gα9*^-^, and *gβ*^- ^cells is not due to low levels of extracellular AprA or CfaD.

### Gα9 and Gβ affect the number of cell surface AprA binding sites

**Figure 4:**
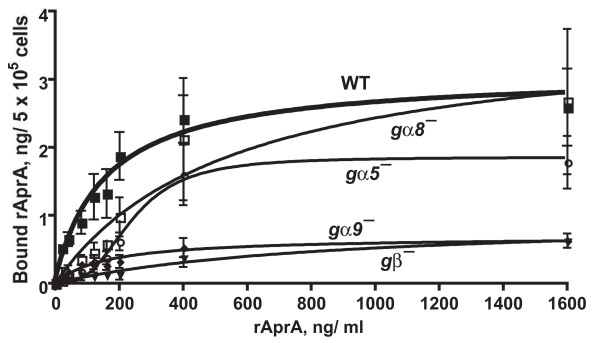
**The binding of recombinant AprA to cells**. The indicated cells (WT is wild-type) were incubated with different concentration of recombinant AprA (rAprA). After 10 min, bound rAprA was quantitated by Western blots (staining for the Myc tag), using known amounts of rAprA as standards. Values are mean ± s.e.m. (*n *= 3). The lines are curve fits to a one-site binding model with no cooperative binding with the exception of the fit to the binding to *gα5^- ^*cells, where the line is a fit to a one-site binding model with a Hill coefficient of 2.9.

We previously observed that wild-type cells show high-affinity saturable binding of rAprA, suggesting that they have an AprA receptor [8]. To determine whether cells that are relatively insensitive to AprA have an observable defect in the ability of rAprA to bind to this receptor, we measured binding of rAprA to cells. Wild-type cells bound rAprA as previously observed (the binding experiments shown here were done in parallel with the experiments presented in [8]). *gα5*^- ^and *gα8*^- ^cells also bound rAprA (Figure 4 and Table 4). The *K*_*D *_and number of binding sites (*Bmax*) for rAprA on *gα5*^- ^and *gα8*^- ^cells were not significantly different by ANOVA from wild-type cells. *gα9*^- ^and *gβ*^- ^cells had significantly reduced numbers of rAprA binding sites, with *K*_*D *_values that were not significantly different from wild-type (Figure 4 and Table 4). Although the binding data for the *gα8^- ^*cells appeared to show a sigmoidal curve, F tests comparing binding with a Hill coefficient of 1 to binding models with a variable Hill coefficient indicated that a model with a Hill coefficient of 1 was preferred for all cell lines except for *gα5^-^*, where a model with a Hill coefficient of 2.9 ± 0.9 (mean ± s.e.m, *n *= 3) was preferred. In addition, F tests to compare a one-site binding model with a two-site binding model indicated that a one-site binding model was the preferred fit for all cell lines. Taken together, the data indicate that cells lacking Gα8 have roughly normal cell-surface rAprA binding, that the lack of Gα5 affects the AprA receptor cooperativity, and that cells lacking Gα9 and Gβ have a reduced number of rAprA binding sites.

**Table 4:** Binding kinetics of rAprA to cells

**Cell type**	**K_D_, *μg*/ml**	**K_D_, nM**	**Bmax, ng/5 × 10^5 ^cells**	**Bmax, molecules/cell**
Wild-type	0.16 ± 0.05	2.7 ± 0.8	3.1 ± 0.4	62,000 ± 8,000
*gα5*^-^	0.54 ± 0.21	9.0 ± 3.5	2.6 ± 0.4	52,000 ± 10,000
*gα8*^-^	0.56 ± 0.28	9.3 ± 4.7	3.8 ± 0.9	76,000 ± 18,000
*gα9*^-^	0.16 ± 0.06	2.7 ± 1.0	0.7 ± 0.1*	14,000 ± 2,000*
*gβ*^-^	0.85 ± 0.33	14.2 ± 5.5	1.0 ± 0.2*	20,000 ± 4,000*

For the indicated cell lines, the K_D _in μg/ml and the Bmax in ng/5 × 10^5 ^cells was obtained from the curve fits in Fig 4. The K_D _in nM and Bmax in molecules/cell were calculated using a molecular mass of 60 kDa for rAprA. All values are mean ± SEM, n = 3. A * after a value indicates that the value was significantly different from the wild-type value with p < 0.05 (1-way ANOVA, Dunnett's test).

### Gα8 and Gβ are required for GTPγS inhibition of rAprA binding

**Figure 5:**
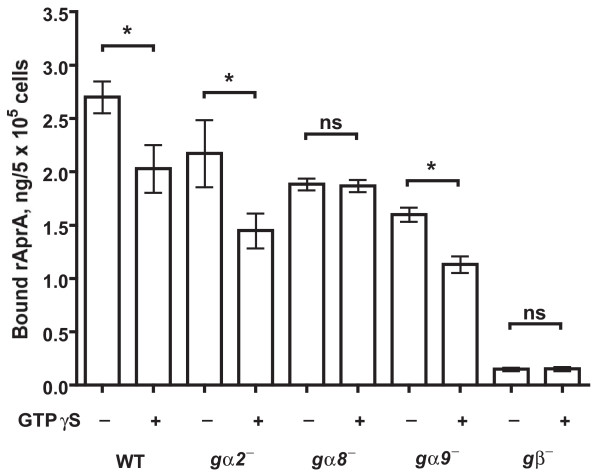
**The effect of GTPγS on recombinant AprA binding to membranes**. Membranes from the indicated strains were incubated with recombinant AprA (rAprA) in the presence or absence of GTPγS. rAprA bound to the membranes was measured as in Figure 4. The presence of GTPγS significantly decreased the binding of rAprA to WT, *gα2*^- ^and *gα9*^- ^membranes in comparison with the buffer control (*, *P *< 0.05), while the presence of GTPγS had no significant effect (ns) on the binding of rAprA to *gα8*^- ^and *gβ*^- ^membranes (paired t-tests). Values are mean ± s.e.m. (*n *= 3).

GTPγS is a non-hydrolyzable analog of guanosine triphosphate that keeps Ga subunits in the GTP-bound active form [11]. Adding GTPγS to membranes affects ligand binding to G protein-coupled receptors [11]. To determine whether the binding of rAprA is affected when Gα subunits are activated, membranes from vegetative wild-type cells were incubated with rAprA in the presence of GTPγS. Wild-type membranes bound rAprA, and GTPγS caused a ~25% decrease in the binding (Figure 5). To identify which Gα subunit is involved in the AprA signal transduction pathway, similar binding assays were done with membranes from cells lacking Gα2, Gα8, Gα9, or Gβ. GTPγS decreased rAprA binding to membranes from *gα2*^- ^and *gα9*^- ^cells, whereas GTPγS had no significant effect on rAprA binding to membranes from *gα8*^- ^and *gβ*^- ^cells (Figure 5). In addition, the binding of rAprA to membranes from *gβ*^- ^cells was strongly reduced when compared with all other cell lines. Together, the data suggest that Gα8 and Gβ are required for the GTPγS-induced decrease in rAprA binding to membranes.

### Gα8 and Gβ are required for AprA-stimulated GTP binding

**Figure 6:**
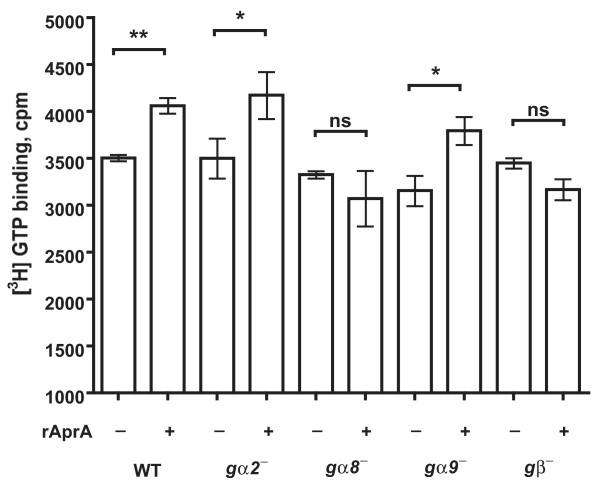
**The effect of recombinant AprA on GTP binding to membranes**. The binding of [^3^H]GTP to membranes in the presence or absence of recombinant AprA (rAprA) was measured following [27]. Wild-type membranes showed significantly increased [^3^H]GTP binding in the presence of rAprA when compared with [^3^H]GTP binding in the absence of rAprA with *P *< 0.01 (**), while *gα2*^- ^and *gα9*^- ^membranes showed increased [^3^H]GTP binding with *P *< 0.05 (*). [^3^H]GTP binding to *gα8*^- ^and *gβ*^- ^membranes was not significantly different (ns) (paired t-tests). Values are the mean ± s.e.m of at least three independent experiments.

Binding of a ligand to a G protein coupled receptor induces the associated Gα protein to bind GTP [33]. To determine whether AprA induces GTP binding, we incubated cells with [^3^H]GTP in the presence or absence of rAprA. The addition of rAprA increased the binding of [^3^H]GTP to membranes from wild-type cells (Figure 6). rAprA also increased the binding of [^3^H]GTP to *gα2*^- ^and *gα9*^- ^membranes. However, rAprA had no significant effect on the binding of [^3^H]GTP to membranes from *gα8*^- ^and *gβ*^- ^cells (Figure 6). These results indicate that Gα8 and Gβ are required for the AprA-induced binding of GTP to membranes.

### Like AprA, Gα8 potentiates spore viability

We previously observed that *aprA^- ^*cells, when starved, form fruiting bodies that have an abnormally low number of spores, and these spores have very poor viability [4]. To determine whether *gα8^- ^*cells have a similar phenotype, we examined spore production and viability (Table 5). Cells lacking Gβ do not aggregate or form spores [25]. We found that *gα8^- ^*cells form roughly normal numbers of spores, but these spores have a significantly reduced viability. This suggests that although both *aprA^- ^*and *gα8^- ^*cells have the advantage of rapid proliferation, they both have the disadvantage of poor spore viability.

**Table 5:** Spore viability

**Cell type**	**Percent of cells forming visible spores**	**Percent of cells forming detergent-resistant spores**
Wild-type	89 ± 8	31 ± 6
*gα8*^-^	75 ± 8	14 ± 5 *

Cells were counted and then starved on filter pads to generate fruiting bodies. After three days, spores were harvested and counted. Spores were then treated with detergent to kill other cells (stalk cells are already dead), washed, and then diluted and plated with bacteria on agar plates. The number of colonies that appeared was then counted to determine the number of viable detergent-resistant spores. All values are mean ± SEM, n = 3. A * after a value indicates that the value was significantly different from the wild-type value with p < 0.05 (t test).

## Discussion

AprA appears to act like a chalone that binds to cell surface receptors and negatively regulates proliferation (the increase in the number of cells per hour) [4]. We found here that, like *aprA*^- ^cells, *gα8*^- ^and *gβ*^- ^cells proliferate rapidly but have normal growth rates (the increase in the cell mass and protein per nucleus per hour), and that cells lacking Gα8 or Gβ have a low sensitivity to rAprA. Like *aprA^- ^*and *cfaD^- ^*cells, *gα8^- ^*and *gβ^- ^*cells die off faster than wild-type after cells reach saturation. Spores tend to be mononucleate, and like *aprA^- ^*and *cfaD^- ^*spores, *gα8^- ^*spores have poor viability (*gβ^- ^*cells do not aggregate or form spores). rAprA increases GTP binding to membranes, and this requires Gα8 and Gβ. Conversely, GTPγS inhibits the binding of rAprA to cells, and this also requires Gα8 and Gβ. Together, the data suggest that AprA uses a G protein signaling pathway to slow proliferation but not growth at high cell density, and that Gα8 and Gβ are components of part of this pathway.

There are four main classes of Gα subunits, G_s_, G_i/o_, G_q_, and G_12 _[34]. G protein pathways that regulate proliferation in response to paracrine signals tend to contain G_i/o_-type Gα subunits [9]. For instance, the neurotransmitter dopamine inhibits forebrain neural stem cell proliferation by binding to the D2 receptor that couples with a G_i/o _protein [35,36]. The sequence of Gα8 has high identity (40%) to many other G_i/o_-like Gα subunits.

Gα8 is expressed in vegetative cells and throughout development, and cells lacking Gα8 form fruiting bodies with a roughly normal morphology [37]. Developing *aprA^- ^*cells have abnormal fruiting bodies and a more severe spore viability defect than *gα8^- ^*cells [4]. In addition, *aprA^- ^*cells have less mass and protein per nucleus than wild-type, whereas *gα8^- ^*cells have roughly normal amounts of mass and protein per nucleus, and *gα8^- ^*cells have a small residual sensitivity to rAprA. These four differences between *aprA^- ^*cells and *gα8^- ^*cells indicate that Gα8 mediates some but not all of the effects of AprA.

Gβ is also expressed in vegetative cells and throughout development, and cells lacking Gβ do not aggregate [25]. Gβ interacts with different Gα subunits to form functional heterotrimeric G protein complexes [25]. Our observation that Gβ is part of the AprA signaling pathway increases the list of different pathways, such as cAMP and folic acid signal transduction, that involve Gβ [38].

Gα9 is expressed during growth and throughout *Dictyostelium *development [24]. During the aggregation stage of development, Gα9 inhibits chemotaxis [24,39]. In addition, Gα9 couples to a GABA receptor, GrlE, to regulate spore differentiation during late development [40]. The rapid proliferation of *gα9^- ^*cells, and their low sensitivity to rAprA, would at first glance suggest that Gα9 is part of the AprA signal transduction pathway. However, three observations suggest that Gα9 is part of a different signal transduction pathway that regulates the response of cells to AprA. First, unlike AprA, CfaD, Gα8, or Gβ, Gα9 appears to inhibit both proliferation and growth. Second, unlike Gα8, Gα9 is not required for AprA-stimulated GTP binding to membranes. Third, again unlike Gα8, Gα9 is not required for the GTPγS-induced decrease in rAprA binding to membranes. Interestingly, although *gα9^- ^*cells have fewer cell-surface rAprA binding sites than wild-type cells, the binding of AprA to membranes from mechanically lysed *gα9^- ^*cells is similar to that of wild-type cells, suggesting the possibility that Gα9 regulates the transport of AprA receptors to the plasma membrane. A second *gβ*-like gene (gpbB, DDB_G0275045) is encoded by the *Dictyostelium *genome [15], so Gα9 may possibly interact with this other Gβ to regulate growth.

Despite having very low extracellular levels of AprA and CfaD, *gα2^- ^*cells have roughly normal proliferation curves, and rAprA slows their proliferation. The latter observation suggests that Gα2 is not part of the AprA signal transduction pathway. One would predict that cells with low extracellular AprA and CfaD would cause rapid proliferation, so it appears that the loss of Gα2 has some deleterious effect on cell proliferation through a mechanism (possibly associated with accumulating less extracellular protein) that does not involve the AprA pathway.

Although the *gα5 *mRNA was not detected in the vegetative cell RNA pool on a Northern blot of RNA from different developmental time points [41], the binding of rAprA to vegetative *gα5^- ^*cells showed cooperativity with a Hill coefficient of ~3, unlike the binding of rAprA to vegetative wild-type, *gα8^-^*, *gα9^-^*, and *gβ^- ^*cells. This suggests that Gα5 is expressed in vegetative cells, and is somehow required for the normal function of the AprA receptor.

## Conclusion

A variety of indirect evidence has led to the hypothesis that some chalones act through G protein-mediated pathways [2]. Our observation that the *Dictyostelium *chalone AprA uses a G protein-mediated signal transduction pathway involving Gα8 strongly supports this hypothesis, and suggests that therapeutics targeting chalone-activated G protein signal transduction pathways may be useful to regulate the proliferation of specific cell types.

## Authors' contributions

Growth curves, proliferation inhibition by rAprA, and rAprA binding assays were carried out by JMC and DB. DB did the AprA and CfaD in conditioned medium Western blots, mass and protein measurements, the GTP binding assays, the GTPγS assays, and the spore viability assays. Nuclei staining and counts were done by NEH. DB and RHG did the experimental design, statistical analysis, and wrote the manuscript. All authors read and approved the final manuscript.
